# Teaching modern pancreatic surgery: close relationship between centralization, innovation, and dissemination of care

**DOI:** 10.1093/bjsopen/zrad081

**Published:** 2023-09-12

**Authors:** Giampaolo Perri, Jony van Hilst, Shen Li, Marc G Besselink, Melissa E Hogg, Giovanni Marchegiani

**Affiliations:** Department of General and Pancreatic Surgery, Verona University Hospital, Verona, Italy; Department of Surgery, Amsterdam UMC, location VU, Amsterdam, The Netherlands; Cancer Center Amsterdam, Amsterdam, The Netherlands; Department of Surgical Oncology, University of Chicago, Chicago, Illinois, USA; Department of Surgery, Amsterdam UMC, location VU, Amsterdam, The Netherlands; Cancer Center Amsterdam, Amsterdam, The Netherlands; Department of HPB Surgery, NorthShore Health System, Evanston, Illinois, USA; Department of Surgical, Oncological and Gastroenterological Sciences (DiSCOG), University of Padua, Padua, Italy

## Abstract

**Background:**

Pancreatic surgery is increasingly moving towards centralization in high-volume centres, supported by evidence on the volume–outcome relationship. At the same time, minimally invasive pancreatic surgery is becoming more and more established worldwide, and interest in new techniques, such as robotic pancreatoduodenectomy, is growing. Such recent innovations are reshaping modern pancreatic surgery, but they also represent new challenges for surgical training in its current form.

**Methods:**

This narrative review presents a chosen selection of literature, giving a picture of the current state of training in pancreatic surgery, together with the authors’ own views, and in the context of centralization and innovation towards minimally invasive techniques.

**Results:**

Centralization of pancreatic surgery at high-volume centres, volume–outcome relationships, innovation through minimally invasive technologies, learning curves in both traditional and minimally invasive surgery, and standardized training paths are the different, but deeply interconnected, topics of this article. Proper training is essential to ensure quality of care, but innovation and centralization may represent challenges to overcome with new training models.

**Conclusion:**

Innovations in pancreatic surgery are introduced with the aim of increasing the quality of care. However, their successful implementation is deeply dependent on dissemination and standardization of surgical training, adapted to fit in the changing landscape of modern pancreatic surgery.

## Introduction

Pancreatic surgery is a low-volume, but high-complexity, field, with a daunting learning curve and challenging morbidity from an individual and system level. Pancreatoduodenectomy (PD) is still burdened by 20 per cent major morbidity, 12 per cent failure to rescue (FTR), and 2 per cent in-hospital mortality rates at tertiary referral centres^1^. This review attempts to highlight key issues in improving the quality of pancreatic surgery via training in the context of modern practice, which is increasingly moving towards innovations, such as centralization and minimally invasive surgery. The first part will describe the current evidence supporting centralization, discussing its pros and cons, and minimally invasive pancreatic surgery, with a particular focus on robotic PD (RPD), as one of the main innovations in the field. The second part will then focus on the current state of training in pancreatic surgery, including the impact of innovations on technical aspects, and how to standardize and disseminate training globally.

## Innovations in pancreatic surgery

### Centralization

Pancreatic surgery is associated with high postoperative morbidity and an extensive amount of research is performed to investigate strategies to lower these rates. In 2002, Birkmeyer *et al*.^2^ described the importance of hospital volume in lowering mortality after surgery in the USA, across 14 different surgical procedures. The volume–outcome relationship was the strongest for pancreatic surgery, where absolute differences in adjusted postoperative mortality rates between very low-volume hospitals (fewer than 1 annually) and high-volume hospitals (more than 16 annually) ranged from over 12 per cent. Birkmeyer *et al*.^3^ additionally showed how 50 per cent of the effect of hospital volume on mortality was explained by the effect of surgeon volume. In 2011, Finks *et al*.^4^ investigated trends of hospital volume over time and the impact of the previously discussed publications. Between 1999 and 2008 hospital volumes for pancreatic resection increased from a median of 5 (interquartile range (i.q.r.) 2–14) to a median of 19 (i.q.r. 5–35) annual cases in the USA. This increase was explained by a 50 per cent increase in patients undergoing pancreatectomy and on the other hand a 25 per cent decrease in the number of hospitals performing this procedure. Postoperative mortality after pancreatic surgery decreased by 19 per cent during the study interval; 67 per cent of this decline was estimated to be explained by the higher hospital volume. This research from the USA showed for the first time the importance of high-volume hospitals and high-volume surgeons to improve postoperative mortality, despite possible confounding factors and bias brought by a data linkage methodology.

In recent years, data from European countries on the volume–outcome relationship in pancreatic surgery increased, as summarized in *Table 1*. Several nationwide database studies were published with an implication to move towards centralization^5–14^. A study from Germany, including over 60 000 patients, showed that mortality (after adjusting for risk factors) ranged from 6.5 per cent (95 per cent c.i. 6.0 to 7.0) in high-volume hospitals up to 11.5 per cent in low-volume hospitals^15^. Moreover, it was estimated that centralization of pancreatic surgery to centres performing at least 20 resections annually could prevent 94 deaths each year in Germany (21 per cent of all deaths occurring in low- and very low-volume hospitals). Also, a study from the Netherlands, including 2155 patients, showed a clear volume–outcome relationship for PD, with significantly higher mortality rates in lower-volume centres (14.7, 9.8, 6.3, and 3.3 per cent in very low-, low-, medium-, and high-volume hospitals respectively, *P* < 0.001)^14^. Data on the volume–outcome relationship for pancreatic surgery are mostly studied in PD. Data for distal pancreatectomy (DP) are scarce and the available data do not show a clear volume–outcome relationship for DP^8,16^. This could be explained by the fact that DP is less complex, with overall lower postoperative mortality rates.

**Table 1:** Selected literature on hospital volume–outcome relationship

Reference	Country	Surgery type	Volume cut-off	Outcome	Outcome (detailed)
**All**					
Birkmeyer *et al*.	USA	Pancreatic resections	16/year	Mortality decreases in high volume	OR 0.20 (95% c.i. 0.14,0.19)
Balzano *et al*.	Italy	PD	104/year	Mortality decreases in high volume	OR 0.20 (95% c.i. 0.08,0.52)
de Wilde *et al*.	Netherlands	PD	20/year	Mortality decreases in high volume	3.3% *versus* 8.6% (*P* = 0.034)
Yoshioka *et al*.	Japan	PD	18/year	Mortality decreases in high volume	OR 0.25 (95% c.i. 0.14,0.43)
Krautz *et al*.	Germany	Pancreatic resections	105/year	Mortality decreases in high volume	OR 0.47 (95% c.i. 0.41,0.54)
van Rijssen *et al*.	Netherlands	PD	30/year	Failure-to-rescue decrease in high volume	OR 3.9 (95% c.i. 1.6,9.6)
Nymo *et al*.	Norway	PD	40/year	Mortality decreases in low–medium volume	OR 0.24 (95% c.i. 0.07,0.82)
Kuemmerli *et al*.	Switzerland	PD	20/year	Mortality decreases in high volume	OR 1.45 (95% c.i. 1.15,1.84)
**Minimally invasive**					
Sharpe *et al*.	USA	Laparoscopic PD	10/year	Mortality decreases in high volume	OR 0.98 (*P* < 0.001)
Adam *et al*.	USA	Minimally invasive PD	22/year	Postoperative complications decrease in high volume	OR 1.74 (95% c.i. 1.03,2.94)
Kutlu *et al*.	USA	PD	25/year	Mortality after laparoscopic PD decreases in high volume	OR 2.70 (95% c.i. 1.06,6.87)
Torphy *et al*.	USA	Minimally invasive PD	6/year	Mortality decreases in high volume	OR 0.70 (95% c.i. 0.51,0.96)

PD, pancreatoduodenectomy.

Most of the research on hospital volume is focused on mortality as the primary outcome. It was often thought that a higher postoperative mortality was caused by a higher complication rate, but more recent evidence shows that the occurrence of complications is less relevant compared with how these complications are managed. The term FTR defines mortality caused by a major postoperative complication^17^. FTR is an indicator of the management of complications and is associated with volume, staffing levels, and technology status^18–20^. A study from the Dutch Pancreatic Cancer Audit, which included all pancreatic resections performed in the Netherlands from 2014 to 2015, investigated the FTR rate between centres with different mortality rates^21^. Variations in mortality rates were attributed to differences in FTR rather than the rate of major complications. Additionally, FTR was independently predicted by a hospital volume of fewer than 30 PD annually (OR 3.9 (95 per cent c.i. 1.6 to 9.6)). The relationship between volume and FTR was also shown in USA data comparing FTR and complications between different centre volumes^22^. This study also showed that the volume–outcome relationship was stronger for FTR compared with complications. These data indicate that high-volume centres are able to rescue patients from complications better than low-volume centres.

Based on clear volume–outcome relationships shown by previous studies, several pledges and different measures for centralization were undertaken in several countries^7–9,12,14,23–26^. The Leapfrog group, a consortium of large corporations and public agencies that purchase healthcare information, established a minimum advised hospital volume of 20 annual pancreatic resections and a surgeon volume of ten annually in the USA^27,28^. In Switzerland, the national regulatory cut-off is set at more than 12 PD annually, which decreased postoperative mortality (OR 1.25 (95 per cent c.i. 0.98 to 1.60))^8^. In 2003, in the Netherlands, the Dutch Pancreatic Cancer Audit was established; this audit is mandatory and all 18 centres performing pancreatic resections are participating^29^. In the Netherlands, the national regulatory cut-off is set at more than 20 pancreatic resections annually. The first analysis after centralization showed a decrease in the in-hospital mortality rate (9.8 to 5.1 per cent, *P* = 0.040)^14^. Also, high-volume centres had higher radical resection rates (OR 0.62 (95 per cent c.i. 0.41 to 0.93)) and improved survival (HR 1.34 (95 per cent c.i. 1.09 to 1.65))^13,30^. Additionally, this Dutch situation offered the opportunity to study the volume–outcome relationship in centres with an annual volume of more than 20. This study also showed a clear volume—outcome relationship for mortality, which persists when also studied in centres performing more than 40 PD annually^13^. Therefore, the threshold for a plateau phase for volume remains unknown. But the current evidence supports at least a threshold of 20 PD annually for centres and therefore supports centralization of pancreatic surgery care.

Despite the overwhelming evidence for centralization, some critics remain^26^. Pancreatic surgery in Norway has been restricted to only five centres for more than a decade, with a longstanding centralization system, despite a large variation in unit volume. A Norwegian study, including 930 patients after PD between 2012 and 2016, did not show a volume–outcome relationship^12^. Mortality rates were low (in-hospital mortality 2 per cent and 90-day mortality 4 per cent) with no differences between regions. However, the five centres were performing pancreatic resections with a volume ranging from a mean of 84 to 513 in the 4-year study interval, so, despite the large difference in annual case load, overall hospital volumes were not low. A more recent analysis, including all PD performed in Norway between 2015 and 2016, showed a similar FTR rate and lower 90-day mortality (OR 0.24 (95 per cent c.i. 0.07 to 0.82)) for low–medium-volume compared with high-volume (at least 40 PDs per year) centres, questioning the utility of centralization beyond medium volume^31^. A possible downside of centralization could be decreasing or differing referral rates because of travel distance to the hospital or a lack of knowledge on pancreatic surgery in the presenting hospital. Interestingly, the Norwegian data showed comparable PD rates per 100 000 inhabitants in the different regions over the study interval, which does not indicate these downsides.

Centralization may not be the answer for all countries. For example, a large healthcare database study from France discussed the downsides of centralization^16^. In this study, in 12 670 patients, mortality was 9.2 per cent after PD. Additionally, a clear association between volume and mortality was seen with cut-offs identified at 16 and 40 PD annually. The study described 483 hospitals performing PD with a median annual volume of only three pancreatectomies, and this number did not increase during the study interval (2007–2012). Centres that performed more than 25 pancreatic resections annually did increase during the study interval, but these centres were not distributed evenly across the country and still 50 per cent of patients were operated on in low-volume centres. Therefore, centralization would imply that over 30 per cent of patients would need to be transferred to a different hospital often in a different region. Besides, the authors of the study questioned if the high-volume centres could handle a 50 per cent increase in patient numbers. Based on these downsides, they concluded that centralization in France was not deemed feasible in this manner.

Moving patients from low-volume centres to high-volume centres does nothing to improve quality of care and outcomes in the low-volume centres. It could therefore be considered as ‘the easy way out’. Instead of improving the quality of care in all hospitals, patients are just redirected to the high-volume hospitals. Also, other factors are relevant: travel distance for patients and their families and the associated travel costs, which could be a problem for patients with a lower socio-economic status; and the increase in case load for high-volume centres, leading to longer waiting lists and overwhelmed staff. Countries that introduce centralization should therefore continue to study the effects and focus on these possible downsides.

What confounds this already complex discussion of volume and centralization is the rise of minimally invasive pancreatic surgery, which brings up additional issues beyond volume and outcomes: surgeon training.

### Minimally invasive pancreatoduodenectomy

In 1994, Gagner and Pomp^32^ first described the laparoscopic approach to PD. Although they deemed the procedure technically feasible, they questioned if the benefit would be as clear as for other laparoscopic procedures. After the first introduction, several observational studies from expert centres were published, showing potential advantages, such as less delayed gastric emptying and shorter hospital stay^33–36^. However, concerns were raised because of an increase in postoperative complication rates, especially in relatively low-volume centres^34,35,37–39^. As volume was already an important topic in open pancreatic surgery, this discussion was also very important to consider in the minimally invasive approach. A registry-based study from the USA showed that a volume of fewer than 22 cases/year for minimally invasive PD was associated with an increase in postoperative complication rates (OR 1.74 (95 per cent c.i. 1.03 to 2.94))^40^. Additionally, it is suggested that the volume–outcome relationship is even stronger for laparoscopic PD (LPD) compared with open PD^36^. Two single-centre RCTs comparing LPD with open PD performed in high-volume expert centres showed a decrease in length of hospital stay and comparable postoperative complication rates^41,42^. LEOPARD-2 was the first multicentre RCT^43^. This trial aimed to investigate outcomes after LPD compared with open PD in medium-volume centres (median of 19 LPD annually). All participating centres were trained in a nationwide training programme for LPD and after performing 20 procedures they were allowed to participate in the trial^44^. Despite the efforts to safely introduce and assess this new technique according to the IDEAL framework^45^, the LEOPARD-2 trial was prematurely terminated because of a difference in 90-day complication-related mortality (5 (10 per cent) of 50 patients in the LPD group *versus* 1 (2 per cent) of 49 patients in the open PD group, risk ratio 4.90 (95 per cent c.i. 0.59 to 40.44), *P* = 0.20). Although the difference was not statistically significant these unexpected findings were worrisome and show the importance of volume and learning curve in these extensive procedures. Recently, a multicentre trial from China in 656 patients from highly experienced centres reported a shorter length of stay and similar short-term morbidity and mortality rates compared with open PD^46^.

In 2019, the first international evidence-based guidelines on minimally invasive pancreas resections were developed in Miami^47^. Existing literature was reviewed to answer several questions regarding minimally invasive pancreatic surgery. These guidelines suggest that minimally invasive PD should be limited to experienced surgeons in high-volume centres due to the long learning curve and the difficulty of the procedure. The guideline committee suggested that surgical societies should mandate centres that perform minimally invasive PD to maintain a prospective database and that trials should be performed in centres that have completed the learning curve. Additionally, the guidelines focused on the influence of surgeon and centre volume on outcomes in minimally invasive pancreatic surgery. Evidence on surgeon volume for minimally invasive pancreatic surgery is lacking, but evidence on centre volume showed an association with morbidity and mortality. Centres that perform more than 20 minimally invasive PD^40^ or more than 20 PD in total^48^ showed a decreased complication rate (OR 1.74 (95 per cent c.i. 1.03 to 2.94)), and an annual volume of more than 10 total PD or minimally invasive PD showed a decreased mortality rate (OR 0.98, *P* < 0.001)^36,49,50^. The guideline committee proposed that centres should participate in prospective registries to have more data available in the future.

Because literature on volume and minimally invasive PD remains scarce, an effort was made to be more informed on the individual opinions of surgeons through a worldwide survey^51^. This survey was completed by 435 surgeons; only 29 per cent (124) of surgeons were performing minimally invasive PD with a median of 12 procedures performed at the time of the survey. The most frequently mentioned reason for not performing minimally invasive PD was a lack of training in this procedure. Superiority for minimally invasive PD was claimed by only 10 per cent of surgeons participating in this survey. Additionally, in 2022, a Delphi survey was performed amongst experts in minimally invasive pancreatic surgery; these experts reached consensus on a minimum annual volume of 10 DP or 50 PD for performing these minimally invasive techniques^52^. These outcomes show that only a minority of surgeons are performing minimally invasive PD and that more evidence is needed to determine which surgeons and centres should perform this procedure and if outcomes are improved compared with the open approach.

## Teaching modern pancreatic surgery

### Learning curve

There is no accepted, standardized definition of a learning curve in pancreatic surgery. Available definitions tend to differ considerably in terms of the necessary number of procedures to reach proficiency, the presence of different phases of the curve itself, the outcomes evaluated to define proficiency, and the influence of possible cofactors like previous training or complexity of procedures. The lack of clear consensus on the exact metrics and how to achieve these metrics remains a compelling argument for analysis of current training protocols worldwide. Available evidence on learning curves for different pancreatic surgeries is summarized in *Table 2*.

**Table 2:** **Learning curves in pancreatic surgery (adapted from Müller *et al***.**^65^**)

Reference	Country	Patients, *n*	Surgeons, *n*	Analysis	Phases, *n*	LC length (procedures, *n*)	Factors
**Open PD**							
Cameron *et al*.	USA	1000	1	Arbitrary	5	2	OT*, BL, LOS*
Coe *et al*.	USA	1210	Multiple	Arbitrary	4	10	Mortality
Ecker *et al*.	USA	303	1	Arbitrary	4	50	POPF*
Fisher *et al*.	USA	162	1	Arbitrary	2	19	OT, BL, LOS*, complications*
Hardacre *et al*.	USA	60	1	Arbitrary	2	30	OT*, LOS*, adjuvant*
Noda *et al*.	Japan	100	1	Arbitrary	2	50	POPF
Park *et al*.	Korea	300	2	Arbitrary	3	50	OT*, BL
Relles *et al*.	USA	686	47	Arbitrary	3	>16	Mortality
Roberts *et al*.	UK	519	8	Statistical/CUSUM	2	50	POPF*
Schmidt *et al*.	USA	1003	19	Statistical/other	2	20	OT*, BL*, complications*
Tsamalaidze *et al*.	USA	93	1	Statistical/CUSUM	4	30	OT*
Tseng *et al*.	USA	650	3	Arbitrary	2	60	OT*, BL*, LOS*
**Laparoscopic PD**							
Choi *et al*.	Korea	171	1	Statistical/CUSUM	3	40	OT*, conversion, POPF, mortality
Dokmak *et al*.	France	68	–	Arbitrary	2	10	OT*
Huang *et al*.	China	98	1	Statistical/CUSUM	3	34	OT*, LOS*
Ke *et al*.	China	–	–	Arbitrary	4	19	OT*, DGE*
Kim *et al*.	Korea	100	1	Arbitrary	3	33	OT*, complications
Kim *et al*.	Korea	119	1	Statistical/CUSUM	2	47	OT
Kuroki *et al*.	Japan	30	1	Arbitrary	3	10	OT*, BL*
Liao *et al*.	Taiwan	12	–	Arbitrary	2	5	OT*, BL
Lu *et al*.	China	120	1	Arbitrary	4	30	OT, BL*
Morato *et al*.	Spain	50	1	Statistical/CUSUM	4	21	OT*, conversion*, complications
Nagakawa *et al*.	Japan	150	3	Statistical/CUSUM	2	20	OT*, BL*
Nieuwenhuijs *et al*.	Netherlands	20	3	Arbitrary	2	10	Anastomotic complications*
Song *et al*.	Korea	500	–	Statistical/CUSUM	4	55	OT*
Speicher *et al*.	USA	56	5	Arbitrary	6	10	OT*, BL*
Tsamalaidze *et al*.	USA	31	1	Statistical/CUSUM	4	20	OT*
Wang *et al*.	China	1029	–	Statistical/CUSUM	4	40	OT*
Wang *et al*.	China	57	1	Statistical/CUSUM	3	11	OT*
Wang *et al*.	China	550	–	Statistical/CUSUM	3	47	OT*
Zhang *et al*.	China	20	–	Arbitrary	2	10	OT, BL, LOS
**Robotic PD**							
Beane *et al*.	USA	380	3	Statistical/CUSUM	2	35	OT*
Boone *et al*.	USA	120	–	Statistical/CUSUM	5	20	OT*
Chen *et al*.	China	60	2	Statistical	2	40	OT*, BL*
Guerra *et al*.	Italy	59	1	Arbitrary	2	20	Conversion
Kim *et al*.	Korea	70	–	Statistical/CUSUM	2	29	OT
Marino *et al*.	Spain	60	1	Statistical/CUSUM	2	25	OT*, BL*
Napoli *et al*.	Italy	70	1	Statistical/CUSUM	2	33	OT*
Rice *et al*.	USA	514	28	Arbitrary	3	80	OT*, complications*
Schmidt *et al*.	USA	40	2	Statistical/other	–	40	OT*
Shi *et al*.	China	450	3	Statistical/CUSUM	3	100	OT*, BL*
Shyr *et al*.	Taiwan	61	2	Statistical/CUSUM	2	20	OT*
Takahashi *et al*.	USA	65	1	Statistical	2	10	OT, complications
Watkins *et al*.	USA, Italy	92	–	Statistical/CUSUM	2	20	OT
Zhang *et al*.	China	20	–	Arbitrary	2	10	OT*
Zhang *et al*.	China	100	1	Statistical/CUSUM	2	40	OT*
Zhou *et al*.	China	41	1	Statistical/CUSUM	2	8	OT
Zwart *et al*.	Netherlands	275	15	Statistical/CUSUM	2	22	OT*
**Laparoscopic DP**							
Barga *et al*.	Italy	30	–	Arbitrary	3	10	OT*, BL*
Barrie *et al*.	UK	25	1	Statistical/CUSUM	2	3	OT*, BL*
de Rooij *et al*.	UK	111	1	Arbitrary	3	30	POPF*, complications*, LOS*
de Rooij *et al*.	Netherlands	201	32	Arbitrary	2	Before/after training	OT, BL, LOS
Dokmak *et al*.	France	165	3	Arbitrary	2	40	OT
Hasselgren *et al*.	Sweden	37	2	Arbitrary	2	18	OT*, complications*
Kim *et al*.	Korea	65	–	Statistical/CUSUM	2	16	Complications
Kneuertz *et al*.	USA	132	–	Arbitrary	2	66	OT*
Liao *et al*.	Taiwan	64	1	Statistical/CUSUM	2	16	OT*
Lof *et al*.	UK	570	12	Arbitrary	4	15	Complications*, ICU admission*, LOS*
Malleo *et al*.	Italy	100	–	Arbitrary	3	33	OT*
Nachmany *et al*.	Israel	39	5	Arbitrary	4	17	OT
Park *et al*.	Korea	26	1	Statistical/other	2	12	OT*
Ricci *et al*.	Italy	32	1	Statistical/other	2	17	OT*
Sahakyan *et al*.	Norway	640	4	Arbitrary	5	80	OT*
**Robotic DP**							
Benizri *et al*.	France	11	5	Statistical/CUSUM	2	7	OT*, conversion, complications, reoperation
Klompmaker *et al*.	USA	80	3	Statistical/CUSUM	2	31	OT
Napoli *et al*.	Italy	55	1	Statistical/CUSUM	2	10	OT
Shakir *et al*.	USA	100	3	Statistical/CUSUM	2	20	OT
Shyr *et al*.	Taiwan	70	2	Statistical/CUSUM	2	37	OT*
Takahashi *et al*.	USA	43	1	Statistical/other	2	5	OT*

* Statistically significant. LC, learning curve; PD, pancreatoduodenectomy; OT, operating time; BL, blood loss; LOS, length of stay; POPF, postoperative pancreatic fistula; CUSUM, cumulative sum; DGE, delayed gastric emptying; DP, distal pancreatectomy.

The range of cases required for a learning curve goes from 7 to 250 for PD and 10 to 40 for DP in the literature, and can become even wider when considering different types of approach (open, laparoscopic, or robotic)^53–60^. For open PD, Tseng *et al*.^61^ published a learning curve of 60 surgeries to improve blood loss, operating room time, length of stay, and R0 resection rate. Roberts *et al*.^62^ similarly identified 50–70 PD to achieve a reduction of pancreatic fistula risk. In a review by Vining and Hogg^63^ the learning curve required for achieving proficiency in laparoscopic DP (LDP) was six to 40 operations and five to 40 operations for robotic DP (RDP). The learning curve for minimally invasive PD was higher (10 to 50 for LPD and 15 to 80 for RPD). A recent study from China shows a three-phase learning curve in a series of 450 RPD, with a first inflection after 100 cases and a conclusion after 250 cases^64^.

Recently, a systematic review analysed learning curves for open and minimally invasive pancreatic surgery, identifying target parameters and defining a three-phase model of learning (competency, proficiency, and mastery)^65^. The number of procedures to achieve the first competency phase of the curve was 30 for open PD, 39 for LPD, 25 for RPD, 16 for LDP, and 15 for RDP. Intraoperative parameters (blood loss, operating time) improved earlier (from competency to proficiency), whereas postoperative parameters (complications, pancreatic fistula) improved later (from proficiency to mastery). Interestingly, oncological outcome parameters were never used to evaluate learning curves in the literature, but, in 21 per cent of the studies assessing them, they were found to change between different learning phases. Interestingly, a recent report from the Netherlands focused on the learning curves for RPD in ‘second-generation’ centres trained in a dedicated nationwide programme (LAELAPS-3)^66^. The feasibility, proficiency, and mastery learning curves were considerably shorter in ‘second-generation’ centres compared with those previously reported from ‘pioneering’ expert centres. The cut-offs were reached at 15 RPD for feasibility (operating time), 62 RPD for proficiency (major morbidity), and 84 RPD for mastery (textbook outcomes), demonstrating the safety and value of a nationwide training programme in centres with sufficient volume. Previous experience in LPD shortened the feasibility (−12 RPD, −44 per cent), proficiency (−32 RPD, −34 per cent), and mastery (−34 RPD, −23 per cent) phases of the learning curve, but did not improve clinical outcomes.

### Training models

**Fig. 1:**
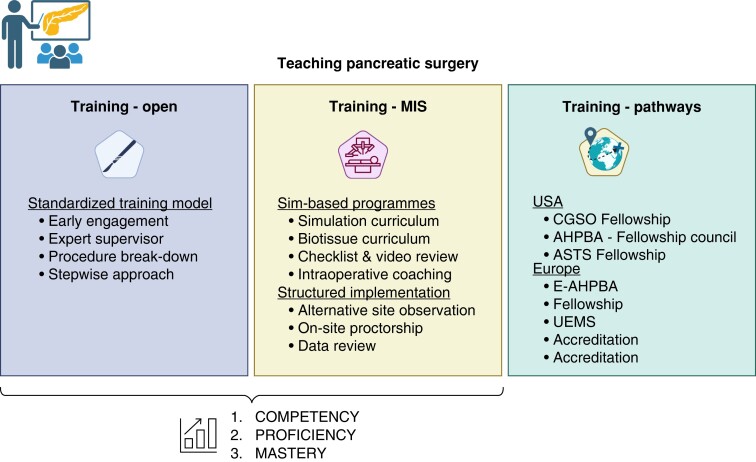
Training pathways and models for open and minimally invasive pancreatic surgery Created using BioRender (https://www.biorender.com). MIS, minimally invasive surgery; Sim, simulation; CGSO, Complex General Surgical Oncology; AHPBA, Americas Hepato-Pancreato-Biliary Association; ASTS, American Society of Transplant Surgeons; E-AHPBA, European–African Hepato-Pancreato-Biliary Association; UEMS, Union Européenne des Médecins Spécialistes.

Training models and pathways for pancreatic surgery are summarized in *Fig. 1*.

#### During residency

The training of hepatopancreatobiliary (HPB) surgeons has changed drastically in the past few decades. Traditionally, pancreas surgery was performed by general surgeons. However, pancreatic surgery is now increasingly performed by surgeons who have completed specialist fellowship training.

In the USA, most residents finishing general surgery residency perform fewer than ten complex HPB operations^67^. Amongst trainees in the USA, the Accreditation Council for Graduate Medical Education (‘ACGME’) showed that a mean of 11.5 pancreatic resections are performed by graduating chief residents^68^. As discussed earlier, hospital volumes vary significantly across the USA. Amongst the 634 hospitals performing PD in the National Cancer Database (‘NCDB’), 49 per cent of the hospitals performed only one procedure/year, whereas 1 per cent performed more than 20 procedures/year^69^. An increased number of training programmes for general surgery and an increased number of fellowship programmes further divide the already small pool of operations. Only five pancreas surgeries are required and likely none of these will be done as a teaching assistant.

In Europe, there are no standardized requirements for general surgery residency, as they are specific for each country. Being not mandatory to be exposed to pancreatic surgery during residency, it is probable that many residents will not be first assistant (or first surgeon) in any pancreatic resection during their training. However, the increasing trend towards sub-specialization for complex surgeries will probably drive an increasing number of residents to earlier, more extensive training opportunities, before applying for a fellowship or a junior faculty position. At the same time, most referral centres for pancreatic surgery are university/teaching hospitals that, in addition to maintaining high standards of patient care, have a duty to provide training for younger trainees.

#### Standardized, stepwise teaching model

**Fig. 2:**
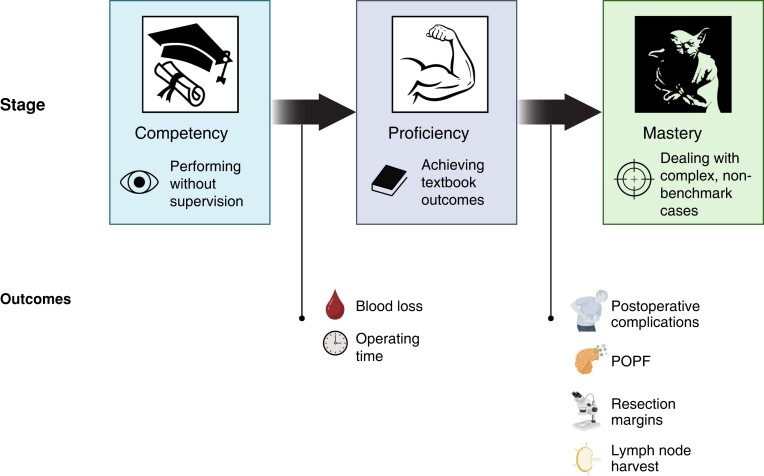
Learning curve stages and outcomes for pancreatic surgery Created using BioRender (https://www.biorender.com). POPF, postoperative pancreatic fistula.

The learning pathway to pancreatic surgery can ideally be divided into at least three distinct phases of variable length^65^ (*Fig. 2*). In the first learning phase, the surgeon will learn to complete pancreatic resections under supervision and assisted by an experienced pancreatic surgeon, with the goal of acquiring ‘competency’. At the end of this phase the surgeon should be able to perform textbook pancreatic resection without the need of supervision, and with progressively diminished operating time (which does not necessarily translate into better patient outcomes yet). In the second phase of ‘proficiency’, the surgeon will become able to solve intraoperative problems of increasing complexity through accumulated experience, finally reaching patient-centred and expert-derived benchmark or textbook outcomes^70,71^. The end of the third and last phase involves reaching ‘mastery’, which implies the ability to operate on more complex non-benchmark cases, such as advanced tumours requiring vascular resections^72^.

The ‘proficiency’ and ‘mastery’ stages require years of experience through consistent repetition and exposure to a huge variety of clinical scenarios, under constant mentoring. They can be ideally imagined as the final goal of formal fellowship training and junior faculty years respectively. However, ‘competency’ in open pancreatic surgery can be safely acquired by younger trainees^73–75^. If approached in a standardized stepwise fashion, a complex procedure such as PD can become the ideal training operation for junior surgical trainees, allowing the development of a wide range of skill sets matching the increasing difficulty of the required steps^73^. Safety and adequacy of this training model, in which residents in general surgery perform major pancreatic resections, has been consolidated over decades and tested with regards to surgical and oncological outcomes at an Italian teaching hospital^74^.

The model ideally requires a team of four surgeons for each procedure: an expert surgeon (at least 50 major pancreatic resections); a postgraduate year (PGY) 5 senior resident; a PGY 3/4 resident; and a PGY 1/2 junior resident. The residents are trained to perform different steps of the procedure as follows: laparotomy/exploration of the abdomen (junior resident); and opening of gastrocolic ligament, Kocher manoeuvre, superior mesenteric vein exposure, common hepatic artery dissection, portal vein exposure, cholecystectomy, and hepatoduodenal ligament dissection (PGY 3/4 and senior resident). When feasible and safe, PGY 5 senior residents can then complete the resection (including dissection of retro portal lamina - right aspect of superior mesenteric artery). Similarly, during the reconstruction phase the senior resident performs the pancreatic anastomosis in case of low-risk for pancreatic fistula, whereas the hepaticojejunostomy is usually performed by the PGY 3/4 resident, and the gastro-enteric anastomosis is usually carried out by the junior resident.

The teaching surgeon is always supervising, ready to take charge in case of more challenging phases, such as a complex vascular ligation, bleeding, or unexpected difficult dissections. The teaching assistant role should always remain proactive, assisting the trainee in each step of the dissection, actively participating in the surgery, anticipating problems, and acting immediately in difficult situations. The crucial role of teaching surgeons is what ensures the safety and reliability in this teaching model, together with an accurate selection of cases. Risk stratification remains key in this scenario as straightforward resections (for example resectable periampullary tumours) should be more likely chosen for trainees, although they might be associated with challenging reconstructions that should be performed by the teaching surgeon (for example soft pancreas with small main pancreatic duct), whereas, vice versa, trainees can safely perform low-risk pancreato-enteric anastomoses^75^.

#### Formal training paradigm in the USA

Modern training for HPB surgery in North America is defined primarily through three fellowship pathways: the Complex General Surgical Oncology (CGSO) fellowship, the Americas Hepato-Pancreato-Biliary Association (AHPBA) fellowship, and the American Society of Transplant Surgeons (ASTS) fellowship^76^.

All residents, to be eligible for boards, must complete a programme called Fundamentals of Laparoscopic Surgery, which includes didactic and technical components. Additionally, recent data suggest that only 68 per cent of surveyed residency programmes have a structured robotic curriculum^77,78^; however, compliance of residents within the curriculum and ability of the residents to sit at the console are still quite low^79^.

- The CGSO fellowship
  A minimum of 240 cancer-related operations must be performed by the CGSO fellow with specific requirements among various surgical oncology specialties. Fellows must also actively participate in multidisciplinary meetings and discussions for at least 120 cancer patients. For hepatobiliary/pancreatic cancer, a minimum of 35 surgeries and 25 multidisciplinary patients are required. For CGSO fellows with a specific interest in HPB training, a subset of CGSO fellowships can offer a sufficient volume for CGSO certification plus the Fellowship Council HPB certificate^80^.
  One CGSO fellowship offers extensive experience with robotic pancreatic resections and an in-depth training programme^81^; however, this programme was not reproducible at other CGSO fellowship sites^82^.
- The AHPBA pathway
  The Fellowship Council is a consortium of sponsoring specialty societies developed to provide fellowship programme accreditations. Currently there are 21 AHPBA-sponsored fellowship programmes. A 2-year programme and 100 HPB cases are required for the HPB certification. Within the 100 HPB cases, a minimum of 25 PD are required along with 5 being minimally invasive resections^53^. Data presented at the State-of-the-Art Conference on Minimally Invasive Pancreas Surgery in Sao Paolo, Brazil showed that AHPBA fellows graduated with a mean of 33 PD (range 13–61) and 17 DP (range 5–33). Fellowship programmes included 17 per cent performing LPD, 83 per cent performing LDP, 25 per cent performing RPD, and 29 per cent performing RDP^83^.
- The ASTS fellowship
  Transplant surgery fellowships offer basic training programmes (kidney and/or liver transplant), as well as specialized transplant fellowship programmes (pancreas, intestine, hepatobiliary, and HPB surgery). The HPB accredited transplant fellowships must complete a minimum of 25 or more major non-transplant-related pancreatic operations, consecutively for no less than 2 years. These operations are defined as PD, total or partial pancreatectomy, and pancreatic drainage operations. Individual fellows must complete a minimum of 50 HPB cases, 15 being non-transplant major pancreatic operations.

#### Formal training paradigm in Europe

Postgraduate training is not standardized in Europe as it is in the USA, but there are opportunities for official accreditation as an HPB surgeon, as well as for accredited fellowship programmes.

- Union Européenne des Médecins Spécialistes (UEMS) accreditation
  The HPB surgery division of the UEMS (https://uemssurg.org/surgicalspecialties/hpb-surgery) organizes European Board of Surgery Qualification (EBSQ) examinations every year, in collaboration with the European–African Hepato-Pancreato-Biliary Association (E-AHPBA). To apply for the EBSQ examination, candidates must have completed surgical training and have a minimum of 2 years of training in HPB surgery after board certification. Candidates must have performed at least 50 major HPB procedures, of which at least 10 are pancreatic resections as first surgeon and 10 as first assistant (and at least 5 minimally invasive). Candidates successfully passing the examination are accredited as Fellow of the European Board of Surgery in HPB Surgery (F.E.B.S. HPB Surg).
- E-AHPBA fellowship
  A training programme accreditation has been recently introduced by the E-AHPBA for European centres. Accredited centres will be acknowledged on the E-AHPBA website for fellowship positions (https://eahpba.org/education-and-training/training-programme-accreditation/current-fellowship-opportunities) and will be closely involved in the development of educational strategies of the E-AHPBA.

### Teaching minimally invasive pancreas surgery

Faced with the dilemma of low-volume pancreatic resections and limited exposure to minimally invasive pancreatic resections, the main challenge for trainees, as well as attending surgeons, is to achieve adequate proficiency. Despite advantages to minimally invasive surgery, the main obstacles to surpassing the learning curve for minimally invasive pancreatic resections include the complexity of surgery, the low volume of pancreatic resections performed by most centres annually, and the lack of clear and accessible training pathways. There are many tactics to engage to address training and the learning curve, which include virtual reality simulation, inanimate biotissue simulation, procedural checklist, video review of surgery, intraoperative coaching, alternative site observation, on-site proctorship, and data review.

#### Structured simulation-based technical training programmes

The University of Pittsburgh developed a five-step robotic pancreas curriculum, including: a proficiency-based virtual reality simulation curriculum; a biotissue curriculum; an HPB video library; intraoperative coaching; and skill maintenance with ongoing outcome assessments^84^.

- Virtual reality simulation curriculum
  The proficiency-based virtual reality simulation curriculum included a pre-test/post-test experimental design utilizing various tasks to assess technical competency. Trainees who completed the curriculum demonstrated significant improvement in their post-tests. The various simulator tasks are designed and ordered by difficulty. Mean time to completion of the curriculum was 4.5 h^85^. This is the best starting point for surgeons without previous robotic experience; however, it is potentially unnecessary for surgeons with extensive robotic experience in general surgery, but not in pancreatic resections.
- Inanimate biotissue simulation curriculum
  The biotissue curriculum uses artificial organs and incorporates practice training on the technical aspects of pancreatic anastomosis (hepaticojejunostomy, gastric-jejunostomy, and pancreatojejunostomy), which is designed to teach the steps of the procedure, improve visual cues due to the lack of haptic feedback, and improve technical skills. In a study of CGSO fellows from the University of Pittsburgh, the biotissue anastomosis was graded by two HPB trained surgeons and the study found decreased time for completion, decreased number of errors, and improved modified Objective Structured Assessment of Technical Skills (OSATS)^86^. Suturing is critical for minimally invasive pancreatic resections even without reconstruction to handle bleeding, etc. and this curriculum led to proficiency. In Europe, biotissue was used to compare different minimally invasive techniques. Pooled data from two RCTs with 60 participants from 11 countries compared hepaticojejunostomy and pancreatojejunostomy anastomosis in biotissue using 3D robotic surgery, 3D laparoscopy, or 2D laparoscopy. Primary outcomes were the OSATS and the operating time required to complete both types of anastomosis. Robotic surgery resulted in a higher OSATS score (50, 43, and 39, *P* < 0.010) and shorter operating time (56.5, 65.0, and 81.5 min, *P* < 0.001) compared with 3D or 2D laparoscopy^87^.
- Procedural checklist and video review
  Breaking down complex operations into discrete steps is beneficial for teaching a long procedure. For example, the RPD resection has been broken down into the following steps: mobilization, portal dissection, pancreatic neck dissection, uncinate process dissection, and gallbladder removal. The University of Pittsburgh HPB video library contains hundreds of minimally invasive pancreatic resections, broken down into key steps. Video review helps prepare trainees before the operating room on anatomy and the procedural checklist. In addition, video review can be used to assess technical skills and complications^88^.
- Intraoperative coaching
  The concept of coaching is intuitive for training residents, fellows, and junior partners at the same institution. To address trainee readiness for independent practice, various types of coaching for residents and/or attendings have been created, such as video-based training, as well as peer coaching. Surgical coaching assesses surgical skill measures, patient safety, and operating time as a performance metric^89–93^. Surgical coaching can also be adapted in minimally invasive pancreatic resections. In 2012, a procedure-specific training programme for RDP was implemented at a single institution. This programme was designed for practicing pancreas surgeons without prior minimally invasive pancreatic resections experience, with the goals of improving short-term outcomes while maintaining patient safety. The study evaluated five different domains: safety, efficiency, morbidity, oncological efficacy, and cumulative treatment burden. The team was constructed of the surgeon learner, the surgeon coach, and resident trainees. The surgeon coach was a robot-credentialed HPB surgeon. The surgeon learner was encouraged to rotate to the surgeon console as often as possible. There was also the combination of didactics, team training, dry lab preparation, virtual reality simulation, and video review with the surgeon coach. Compared with ‘before training’, RDP performed ‘after training’ resulted in reduction in length of stay, blood loss, and transfusion requirements, whereas morbidity and oncological efficacy were unaffected. The study also demonstrated that the initial learning curve was achieved after 16 cases. After 66 cases, the residents became the first assist and the role of the surgeon coach diminished^94^.

#### Structured implementation programmes (alternative site observation, on-site proctorship, and data review)

- LAELAPS-1
  Structured implementation programmes on a national level are rare. According to the Dutch Pancreatic Cancer Group (DPCG), from 2005 to 2013, only 10 per cent of DP were performed via a minimally invasive approach with one-third being converted to open surgery. Within the 9-year interval there was no significant increase in the use of LDP^95^. To test the feasibility and impact of outcomes on a national training programme, the DPCG developed the LAELAPS-1 nationwide training programme aimed at safe nationwide implementation of LDP. This was a multicentre prospective programme in all 17 Dutch pancreatic centres (each performing at least 20 PD/year). Participating surgeons received detailed technique descriptions, video training, and on-site proctoring. The technical description included a list of surgical instruments, as well as detailed operative explanation, with tips and tricks for intraoperative problems. The study resulted in a 7-fold increase in the use of LDP (9 to 47 per cent), as well as decreased conversion rates, blood loss, and length of hospital stay. After this programme, more pancreatic ductal adenocarcinomas and larger tumours were operated on with the laparoscopic approach^96^. To follow-up the LAELAPS training programme, the multicentre randomized controlled LEOPARD-1 trial compared LDP with open DP. Of note, surgeons were only allowed to participate in the LEOPARD trial after completing the LAELAPS training. The LEOPARD trial included 108 patients from 14 centres between April 2015 and March 2017; 51 patients were randomized to LDP and 57 were randomized to the open group. Patients who underwent LDP had shorter time to functional recovery, less operative blood loss, and lower delayed gastric emptying. The 90-day mortality did not differ significantly between the groups. However, quality of life was better in the minimally invasive DP group^97^. Similarly, the Swedish LAPOP trial also demonstrated that minimally invasive DP resulted in a shorter hospital stay and less operative blood loss compared with open DP^98^.
- LAELAPS-2
  In 2014, the DPCG launched the LAELAPS-2 training programme, which aimed to safely introduce LPD in a prospective multicentre programme. Eight surgeons from four centres completed the programme. All participants had completed the LAELAPS-1 training programme. The entire surgical team of two surgeons performing LPD was held constant during the trial. The operations were proctored by an international expert and later by two Dutch surgeons. The implemented training programme (LAELAPS-2) resulted in an acceptable 11 per cent conversion rate, 15-day median hospital stay, and 4 per cent complication-related 90-day mortality rate. This programme also included technique description and video training with proctoring^44^. Hereafter, the LEOPARD-2 trial followed the LEOPARD-1 trial. As mentioned previously, this multicentre randomized trial aimed at assessing open PD *versus* LPD and was closed early due to safety concerns from increased mortality (that had not reached statistical significance) in the laparoscopic arm at interim analysis^43^. Previously, the PLOT trial from India and the PADULAP trial from Spain had shown acceptable morbidity and mortality with an improved length of stay in the LPD arm. Despite the training done in LAELAPS-2, centres that performed 20 PD/year were performing very few LPD during the LEOPARD-2 trial once inclusion criteria were met, and patients underwent 1 : 1 randomization. This highlighted the volume issue for minimally invasive PD and led to centralization as discussed above to increase volume in accordance with an international consensus.
- LAELAPS-3
  After the early termination of the LEOPARD-2 trial, the DPCG started the LAELAPS-3 multicentre training programme for RPD in centres performing at least 50 PD annually. This was a combination of the structured simulation-based technical training programme from the University of Pittsburgh and the structured implementation of the LAELAPS programme^99^. This programme included virtual reality simulation, inanimate biotissue simulation, procedural checklist, video review of surgery, alternative site observation, increased complexity of RPDs, on-site proctorship, and data review. Now, over 750 RPD have been performed in the Netherlands in the past 6 years and a similar programme has expanded through Europe.
- LEARNBOT
  After the culmination of LAELAPS-3 in 2020, LEARNBOT was developed by the European Minimally Invasive Pancreatic Surgery (E-MIPS) group utilizing the same training strategy as LAELAPS-3 in collaboration with the E-AHPBA. This programme aims to determine the impact of a European training programme for RPD using video, virtual reality simulation, and biotissue anastomoses on clinical outcomes in 20 high-volume centres, defined as centres performing at least 50 PD (both open and minimally invasive) annually. Outcomes are being registered in the ongoing E-MIPS registry. Programmes entering LEARNBOT are selected in accordance with a recent international Delphi consensus study^52^. Experienced surgeons from the Netherlands who have completed the LAELAPS-3 programme and several experienced RPD proctors from across Europe are now serving as proctors. After training, centres are expected to perform at least 20 minimally invasive PD annually and enter their data in the E-MIPS registry. To date over 80 RPD have been performed at 14 trained centres from six countries. In addition, in Europe, the DIPLOMA-2 randomized trial is currently comparing minimally invasive *versus* open PD in a 2 : 1 fashion. Only experienced centres, who have performed at least 60 minimally invasive PD, are included; results are expected in early 2024.

## Conclusion

The past decade has been characterized by increased utilization of minimally invasive pancreatic surgery and growing interest in the volume–outcome relationship. However, there are limited data on how to optimally incorporate centralization and minimally invasive surgery. These changes are happening independently in many countries all over the globe, without any uniformity of practice or clear outcomes, and they may represent challenges as much as opportunities. Their successful implementation is deeply dependent on dissemination and standardization of training. In the case of minimally invasive surgery, participation in a structured training programme is strongly advised. Hospitals, countries, and continents can all learn from the collective and independent experiences of each other. This approach may contribute to standardizing techniques and outcomes, while shortening the learning curve, making, at the same time, optimal training accessible outside of extremely high-volume centres.

## Data Availability

Not applicable.
